# The influence of vibratory massage after physical exertion on selected psychological processes

**DOI:** 10.3389/fpsyg.2024.1380282

**Published:** 2024-05-03

**Authors:** Krzysztof Wrześniewski, Tomasz Pałka, Jan Blecharz

**Affiliations:** ^1^Department of Psychology, Institute of Social Sciences, Faculty of Physical Education and Sport, University of Physical Education, Krakow, Poland; ^2^Department of Physiology and Biochemistry, Institute of Biomedical Sciences, Faculty of Physical Education and Sport, University of Physical Education, Krakow, Poland

**Keywords:** vibrotherapy, cognitive functions, post-exercise recovery, muscle pain, mood states

## Abstract

Good mental preparation of an athlete plays an important role in achieving optimal sports results. An athlete who enters a competition should not feel fatigue resulting from intense physical exercise. Therefore, new and effective methods are being sought that could help accelerate the process of both physical and mental regeneration. Vibrotherapy is one of them. The aim of the study was to determine the optimal frequency of vibration, its duration and the position in which the subjects were placed during the treatments, in relation to the reduction of subjectively perceived exertion muscle pain, mental discomfort, emotional states and the level of cognitive processes that were disturbed by intense physical activity. Sixteen healthy male volunteers were involved in this study. The participants were assessed for their aerobic and anaerobic capacity. Each of the subjects performed a set of intensive physical exercises and then underwent vibrotherapy treatment. In random order, each of the men tested the effectiveness of eight combinations of frequency, duration, and body position. Psychological tests were conducted for each combination: frequency, duration of treatment, and position during treatment, in four stages: (1) before the start of the experiment (baseline POMS measurements), (2) immediately after the exercise (VAS scale, scale examining psychological discomfort and STROOP test), (3) immediately after the vibration treatment (POMS measurements, VAS scale, scale examining psychological discomfort and STROOP test), (4) 24 h after the vibration treatment (VAS scale examining subjective assessment of perceived pain and psychological discomfort). Based on the results, it was concluded that all the studied variables improved significantly over time (after the vibration treatment and 24 h after training). In addition, a statistically significant interaction measurement × frequency was noted for vigor scale (52HZ favored greater improvement in this state), and a statistically significant interaction was found for measurement × time for the VAS scale (*p* < 0.05) – the lower pain value was indicated 24 h after the 10-min vibration treatment. The type of frequency used, position, and duration of the treatment did not play a statistically significant role in changing STROOP test results and severity of psychological discomfort (*p* > 0.05).

## 1 Introduction

In recent years, vibration treatments have become increasingly popular. This has aroused interest in the scientific world, which has contributed to an increasing number of studies on the effectiveness of vibration treatments both somatic (Aminian-Far et al., 2011; Lau and Nosaka, 2011) and psychological (Van Heuvelen et al., 2021). The evidence to date on vibrotherapy suggests that its effects are like those of physical activity such as running, aerobics and other forms of exercise (Yu et al., 2021). Positive effects have been reported on the cardiovascular and musculoskeletal systems and the cognitive system through activation of the central nervous system associated with attention, memory, emotions and learning, such as the prefrontal cortex and amygdala (Regterschot et al., 2014). Previous studies suggest that the effect of vibration treatment depends on factors such as posture and muscle tension, as well as the frequency, amplitude, direction of the MV and duration of exposure (Jordan et al., 2005).

Previous research indicated that the vibration treatments can be used in sports practice, for example, as an alternative method supporting the process of mental regeneration after intense physical exercise. This type of physical effort is a big stressor not only modifying the functional state of the human body (Laing et al., 2008) but it also influences emotional state (Uher, 2018) and subjective muscle pain sensations (Kress and Statler, 2007). How quickly an athlete returns to an optimal mental and physical state after a sports performance (e.g., after a tennis match) may determine its outcome during the next performance (e.g., the next day). By mental state we mean the mood states (Lochbaum et al., 2021), the subjective assessment of physical and mental discomfort (Pettersen et al., 2020) and the functioning of cognitive processes (Vestberg et al., 2017; Policastro et al., 2018; Hernández-Mendo et al., 2019). Previous research on vibration treatments focused on its effect, without considering factors such as the use of different vibration frequencies, the body position in which the treatment is performed and its duration [ex: Regterschot et al. (2014), Amonette et al. (2015), and Boerema et al. (2018)]. Therefore, it seems appropriate to ask whether the impact of such treatments may depend on the vibration massage procedure used.

Vibration treatments can be divided into two types: whole body vibration (Morel et al., 2017) and locally applied vibration (Piotrowska et al., 2021). Both methods show positive impact on the human body (Burke and Schiller, 1976; Piotrowska, 2022), emotion regulation, pain perception (Li et al., 2023) and cognition processes (Freitas et al., 2022).

It should be noted that previous research has not focused on this specific aspect, which is the impact of vibrotherapy used to accelerate post- exercise mental restitution. In the field of physiology such research has been done by Pałka et al. (2023), where they indicated that the optimal treatment that can accelerate the process of the redistribution and elimination of pro-inflammatory molecules, the inhibition of local inflammation, and, consequently, the acceleration of the body’s readiness to make another effort should be based on lower ranges of frequency values (2–52 Hz rather than 82–100 Hz). Body position during vibrotherapy had an impact on the results as well - the procedure performed with raised feet gave better results by increasing drainage and elimination of inflammatory components. Also, the duration of the treatments gave different results - for assessing the level of oxygen saturation, a 10- min treatment would be more beneficial. However, to improve the overall work, and a number of biochemical markers, a 45- min treatment is a better choice. Considering that different physiological results can be obtained using different vibration treatment protocols, we wanted to check whether analogous differences could be observed in the case of mental states.

The aim of the present study was to determine the optimal vibration frequency, its duration, and the position in which the subjects were placed during the treatments, in relation to the reduction of perceived pain, mental discomfort, emotional states and the level of cognitive processes disturbed by intensive physical exertion. To achieve our goal, four research questions were posed regarding the optimal (1) frequency (low/high) of the vibration treatment, (2) its duration (10 min/45 min) and (3) body position during the treatment (lying down/lying with slightly raised legs) in order to improve the mood states, and concentration and reduce perceived muscle pain and mental discomfort. We would like to emphasize that the subject of the research was not to indicate the impact of vibration treatments on mental states, which had already been presented in previous studies (see, for example: Rasti et al., 2020; Serritella et al., 2020; Souza Freitas et al., 2022). This study focused only on selecting the optimal frequency, body position and duration of treatments aimed at accelerating post-exercise mental regeneration.

## 2 Materials and methods

The study involved 16 healthy men with current medical examinations who met the following inclusion criteria: non-athletic trainees with similar levels of physical fitness (ACSM). Exclusion criteria included any contraindications to physical activity and contraindications to the use of vibration (Piotrowska et al., 2021). The following inclusion criteria were applied: male sex, age between 20 and 30 years, and body composition parameters within the normal ranges for the Polish population (Jarosz et al., 2020). The exclusion criteria included any contraindications to physical activity and contraindications to the use of vibration (Piotrowska, 2022). During research, the subjects did not consume stimulants or use vitamins or supplements and did not change their dietary habits. All participants were students of physical education and performed physical activities related to the study program. The acceptable additional physical effort included a maximum of 2 h of moderate-intensity activities per week. The average age of the respondents was 22.17 ± 1.4 years. The basic anthropometric characteristics and physiological indicators determined in the aerobic and anaerobic tests were presented in publication of physiological-biochemical team (Pałka et al., 2023).

The research project was approved by the Bioethics Committee of the PMWSZ in Opole No. KB/56/N02/2019. The men were informed, in accordance with the requirements of the Declaration of Helsinki, about the purpose of the study, the methodology used, possible side effects and the possibility of withdrawing from the study at any time without giving a reason. A written informed consent was obtained from the participant for taking part in the study. The entire experiment was conducted under the supervision of medical personnel and a doctor.

The psychological examinations were performed as part of a broader physiological-biochemical research. The examinations took place in the air-conditioned Laboratory of Physiological Basis for Adaptation, using the ISO 9001-certified testing equipment of the Laboratory, which is part of the Central Scientific and Research Laboratory of the University of Physical Education in Krakow. All tests were performed in the morning, at least 2 h after a light meal, to take into account circadian rhythm. Vibrotherapy treatments were conducted with vibrating devices manufactured by Vitberg (Poland). The product is a class IIa medical device, certified by the recognized body of TUV Rheinland (CE0197). In the vibrotherapy treatments, two frequency ranges were selected for testing: I - with low frequency (2–52 Hz) and II - with higher frequency (82–100 Hz), stimulus exposure time - T1 10 min and T2 45 min, respectively, and also two body positions during the massage from the lumbar region to the feet - lying position A, and with the lower limbs raised by 20° - lying position B.

Each participant in random order was subjected to all combinations of vibration massage treatments during the restitution period: I-T1-A, I-T2-A, I-T1-B, I-T2-B, and II-T1-A, II-T2-A, II-T1-B, II-T2-B. Each combination was tested after an anaerobic exercise at 10-day intervals. The interval time was necessary to allow any effects of vibration and exercise to dissipate. In total we gathered 128 sessions results (8 sessions per subject x 16 subjects).

Four test were used as measurement tools: (1) The POMS questionnaire (McNair et al., 1971) in the Polish adaptation by Dudek and Koniarek (1987), the VAS scale (Gift, 1989) investigating (2) subjective assessment of perceived pain and (3) psychological discomfort, and (4) the STROOP test – version S10 – color-word interference, touch screen (Schufried, 2017).

The POMS questionnaire is a self-description tool consisting of 65 items to which the respondent is asked to refer on a five-point Likert scale (0–4). This questionnaire measures the intensity of six emotional states: tension-anxiety, depression-dejection, anger-hostility, fatigue, confusion, and vigor. The measurement can refer to any specified period of time. In the case of this research, respondents were asked to indicate their state “here and now.”

The VAS scale scores are based on self-reported measures of perceived pain symptoms that are recorded with a single handwritten mark placed at one point along the length of a 10-cm line that represents a continuum between the two ends of the scale—“no pain” on the left end (0 cm) of the scale and the “worst pain” on the right end of the scale (10 cm) (Gift, 1989). The VAS scale can be used to evaluate other states (Jamison et al., 2002), in this research we also used it to evaluate psychological discomfort similarly to the pain scale – the left end of the scale – “no psychological discomfort” and the “worst psychological discomfort” on the right end of the scale.

Standardized instrumental tests by Schuhfried (2013) – Vienna Test System were applied to conduct STROOP test which measure respondent’s information processing and attention processes. The STROOP test is a neuropsychological test extensively used to assess the ability to inhibit cognitive interference that occurs when the processing of a specific stimulus feature impedes the simultaneous processing of a second stimulus attribute, well-known as the Stroop Effect (Scarpina and Tagini, 2017). This test measures both speed in reading words and naming colors and speed under conditions of color/word interference (Schufried, 2017).

**Figure 1:**
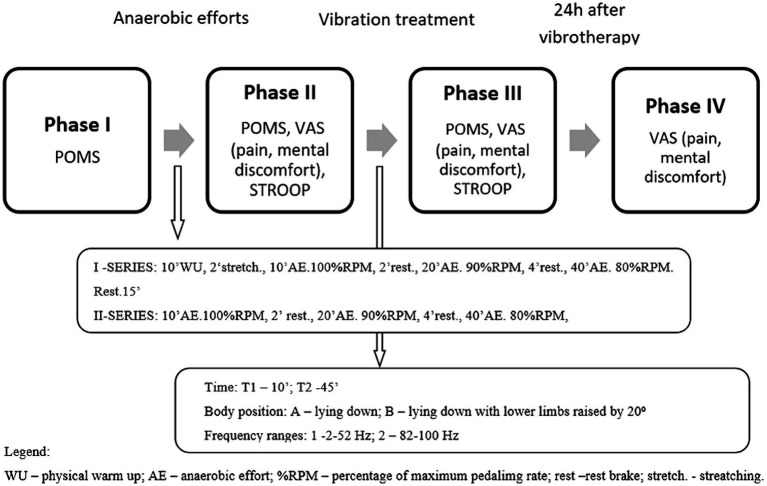
Schematic of the course of the study.

Psychological tests were conducted for each combination: frequency, duration of treatment, and position during treatment, in four stages: (1) before the start of the experiment (baseline POMS measurements), (2) immediately after the exercise (VAS scale, scale examining psychological discomfort and STROOP test), (3) immediately after the vibration treatment (POMS measurements, VAS scale, scale examining psychological discomfort and STROOP test), (4) 24 h after the vibration treatment (VAS scale examining subjective assessment of perceived pain and psychological discomfort) (Figure 1).

### 2.1 Statistics

A two-factor MANOVA analysis of variance (Kim, 2014) was used to examine the interaction between the repeated-measurement factor and intergroup factors (frequency, treatment duration and position). To determine the effect size, a partial eta square (η2P) was calculated, with values >0.01, 0.06, and 0.14 corresponding to small, medium, and large effect size (Miles and Shevlin, 2001). When significant interactions were found, probabilities were calculated for post-hoc tests using the LSD test (Agbangba et al., 2024).

## 3 Results

### 3.1 Profile of mood states (POMS)

The analysis of the vibration treatment effect on the subjects’ emotional states (observed after physical exertion) indicated that regardless of the form of treatment, negative emotional states were reduced: tension-anxiety (*F* (1, 120) = 820.386; *η*^2^_P_ = 0.872; *p* < 0.001), depression-dejection (*F* (1, 120) = 1764.597; *η*^2^_P_ = 0.936; *p* < 0.001), anger-hostility [*F* (1, 120) = 1573.939; *η*^2^_P_ = 0.929; *p* < 0.001], fatigue [*F* (1, 120) = 954.313; *η*^2^_P_ = 0.888; *p* < 0.001] and confusion [*F* (1, 120) = 247.661; *η*^2^_P_ = 0.674; *p* < 0.001]. No effect of vibration frequency, position or treatment duration on the negative emotional was found (*p* > 0.05).

However, a positive emotional state - vigor improved after the treatment [*F* (1, 120) = 447.809; *η*^2^_P_ = 0.786; *p* < 0.001]. In addition, a statistically significant interaction measurement × frequency was noted for this scale [*F* (1, 120) = 6.907; *η*^2^_P_ = 0.054; *p* = 0.010]. The *post hoc* test results (LSD test) indicated that frequency I (2–52 Hz) favored greater improvement in this state.

It is also worth noting that after the vibration sessions (phase 3), the level of negative moods in the study group was significantly (*p* < 0.05) lower and the level of vigor was higher compared to the initial level (phase 1).

The basic characteristics of the variables discussed here are presented in Table 1.

**Table 1:** Descriptive statistics – POMS.

		I-T1A	I-T1-B	IT2-A	I-T2-B	II-T1-A	II-T1-B	II-T2-A	II-T2-B
Tension-anxiety	Phase I	1.31 ± 0.62							
	Phase II	1.91 ± 0.25	1.94 ± 0.27	1.90 ± 0.25	1.91 ± 0.22	1.87 ± 0.27	1.87 ± 0.25	1.96 ± 0.29	1.93 ± 0.24
	Phase III	0.51 ± 0.40	1.22 ± 0.62	0.78 ± 0.42	0.83 ± 0.35	0.95 ± 0.53	0.77 ± 0.34	0.88 ± 0.32	0.86 ± 0.44
Depression-dejection	Phase I	0.44 ± 0.45							
	Phase II	1.53 ± 0.36	1.50 ± 0.38	1.56 ± 0.33	1.53 ± 0.32	1.53 ± 0.37	1.56 ± 0.34	1.55 ± 0.31	1.53 ± 0.38
	Phase III	0.41 ± 0.34	0.40 ± 0.37	0.48 ± 0.35	0.46 ± 0.35	0.40 ± 0.31	0.43 ± 0.31	0.42 ± 0.34	0.35 ± 0.30
Anger-hostility	Phase I	0.73 ± 0.39							
	Phase II	1.71 ± 0.25	1.68 ± 0.22	1.70 ± 0.28	1.74 ± 0.24	1.71 ± 0.28	1.69 ± 0.29	1.76 ± 0.25	1.71 ± 0.27
	Phase III	0.38 ± 0.28	0.42 ± 0.34	0.53 ± 0.35	0.58 ± 0.36	0.54 ± 0.36	0.41 ± 0.29	0.61 ± 0.40	0.50 ± 0.35
Vigor	Phase I	2.68 ± 0.90							
	Phase II	1.31 ± 0.57	1.32 ± 0.56	1.31 ± 0.58	1.35 ± 0.57	1.30 ± 0.57	1.28 ± 0.57	1.33 ± 0.57	1.30 ± 0.57
	Phase III	3.26 ± 0.93	3.11 ± 0.50	2.85 ± 0.77	4.13 ± 1.90	3.05 ± 0.49	2.61 ± 0.75	2.99 ± 0.71	2.84 ± 0.72
Fatigue	Phase I	1.10 ± 0.91							
	Phase II	2.11 ± 0.41	2.14 ± 0.44	2.08 ± 0.41	2.12 ± 0.47	2.14 ± 0.42	2.15 ± 0.55	2.11 ± 0.44	2.17 ± 0.50
	Phase III	0.88 ± 0.61	0.99 ± 0.58	0.95 ± 0.54	1.12 ± 0.53	1.13 ± 0.54	1.08 ± 0.60	1.11 ± 0.56	1.05 ± 0.60
Confusion	Phase I	1.04 ± 0.46							
	Phase II	1.45 ± 0.35	1.50 ± 0.40	1.41 ± 0.31	1.47 ± 0.32	1.41 ± 0.29	1.45 ± 0.37	1.52 ± 0.38	1.49 ± 0.40
	Phase III	0.78 ± 0.43	0.86 ± 0.72	0.54 ± 0.49	0.92 ± 0.59	0.77 ± 0.50	0.68 ± 0.44	0.87 ± 0.62	0.88 ± 0.63

Variables are shown as mean values ± standard deviation (SD).

Phase: I – baseline, II – immediately after the exercise, III – immediately after the vibration treatment; Frequency of vibrations: I: 2–52 Hz, II: 82–100 Hz; vibrotherapy duration: T1: 10 min, T2: 45 min; body position: A: Lying down with lower limbs raised by 20°, B: lying down.

### 3.2 Pain sensation results

Descriptive statistics for the VAS scale are presented in Table 2. Statistically significant differences were found for subjective assessment of muscle pain sensation between the measurements performed [*F*(1.629, 193.820) = 680.004; *η*^2^_P_ = 0.851; *p* < 0.001]. The LSD test results showed statistically significant differences between all 3 measurements. The respondents indicated the highest values for pain sensations immediately after exercise, followed by vibration treatment, and the lowest values 24 h after treatment.

**Table 2:** Descriptive statistics – VAS measures of perceived pain symptoms and psychological discomfort.

		I-T1A	I-T1-B	IT2-A	I-T2-B	II-T1-A	II-T1-B	II-T2-A	II-T2-B
Perceived muscle pain	Phase II	7.44 ± 2.10	7.90 ± 1.77	7.34 ± 1.31	7.87 ± 1.6	7.81 ± 2.04	7.66 ± 1.21	7.31 ± 1.53	6.84 ± 1.76
	Phase III	3.75 ± 2.02	4.20 ± 1.90	4.22 ± 1.84	4.03 ± 2.05	3.97 ± 2.19	3.81 ± 1.71	3.72 ± 1.57	3.72 ± 1.97
	Phase IV	1.75 ± 1.20	1.77 ± 1.27	1.78 ± 1.03	2.44 ± 1.38	1.31 ± 1.03	1.97 ± 1.23	2.56 ± 1.33	2.02 ± 1.19
Psychological discomfort	Phase II	4.94 ± 2.29	5.62 ± 1.78	5.44 ± 2.25	6.09 ± 1.66	5.06 ± 2.02	5.97 ± 2.05	5.56 ± 2.22	5.06 ± 2.20
	Phase III	2.81 ± 2.20	2.91 ± 1.37	3.37 ± 2.22	3.66 ± 1.37	2.66 ± 1.76	3.31 ± 1.99	2.94 ± 2.02	3.03 ± 1.88
	Phase IV	1.09 ± 1.04	1.28 ± 1.06	1.00 ± 0.82	1.00 ±1.10	1.16 ± 1.03	1.00 ± 0.98	1.37 ± 1.02	1.00 ± 0.98

Variables are shown as mean values ± standard deviation (SD).

Phase: II – immediately after the exercise, III – immediately after the vibration treatment, IV – 24 h after the vibration treatment; Frequency of vibrations: I: 2–52 Hz, II: 82–100 Hz; vibrotherapy duration: T1: 10 min, T2: 45 min; body position: A: Lying down with lower limbs raised by 20°, B: lying down.

A statistically significant interaction was found for measurement × time for the VAS scale [*F* (1.629, 193.820) = 4.003; *η*^2^_P_ = 0.033; *p* = 0.027] – the *post hoc* test showed that the lower muscle pain value was indicated 24 h after the 10-min vibration treatment. No significant differences were found for other measurements (*p* < 0.05).

No significant differences were found between frequency and position and the level of perceived pain (*p* > 0.05).

### 3.3 Mental discomfort results

The analysis of the subjects’ perceived psychological discomfort showed statistically significant differences between the results obtained from the performed measurements [*F*(1.576, 189.070) = 338.319; *η*^2^_P_ = 0.738; *p* < 0.001]. The LSD test indicated statistically significant differences between all 3 measurements. Respondents experienced the greatest psychological discomfort immediately after exercise, followed by vibration treatment, and the lowest values were indicated 24 h after treatment (Table 2).

The type of frequency used, the position during the treatment and the duration of the treatment did not play a statistically significant role in changing the severity of psychological discomfort (*p* > 0.05).

### 3.4 The STROOP test results

The analysis of the STROOP test indicated a statistically significant improvement in the quality of task performance after vibration treatment as compared to performance immediately after exercise for the following scales: tendency for reading interference (word-level concentration) [*F* (1, 120) = 20.067; *η*^2^_P_ = 0.143; *p* < 0.001], tendency for interference in naming (non-word-level concentration) [*F*(1, 120) = 119.373; *η*^2^_P_ = 0.499; *p* < 0.001], reading reaction time (baseline) [*F* (1, 120) = 1156.282; *η*^2^_P_ = 0.906; *p* < 0.001], reaction time naming (baseline) [*F* (1, 120) = 739.388; *η*^2^_P_ = 0.860; *p* < 0.001], reaction time reading (interference) [*F* (1, 120) = 531.210; *η*^2^_P_ = 0.816; *p* < 0.001] and reaction time naming (interference) [*F* (1, 120) = 7.291; *η*^2^_P_ = 0.056; *p* = 0.008]. The basic characteristics of the variables discussed here are presented in Table 3.

**Table 3:** Descriptive statistics – STROOP test.

		I-T1A	I-T1-B	IT2-A	I-T2-B	II-T1-A	II-T1-B	II-T2-A	II-T2-B
tendency for reading interference (s)	Phase II	0.13 ±0.06	0.13 ±0.06	0.14 ±0.05	0.13 ±0.08	0.13 ±0.07	0.13 ±0.06	0.13 ±0.07	0.13 ±0.04
	Phase III	0.10 ±0.07	0.10 ±0.07	0.11 ±0.08	0.10 ±0.07	0.10 ±0.06	0.11 ±0.07	0.11 ±0.10	0.10 ±0.07
tendency for interference in naming (s)	Phase II	0.24 ±0.10	0.24 ±0.10	0.24 ±0.10	0.24 ±0.10	0.24 ±0.10	0.24 ±0.10	0.24 ±0.10	0.24 ±0.10
	Phase III	0.16 ±0.11	0.16 ±0.10	0.16 ±0.11	0.15 ±0.11	0.16 ±0.11	0.17 ±0.08	0.16 ±0.11	0.16 ±0.07
reaction time – reading (s) {baseline}	Phase II	0.66 ±0.05	0.65 ±0.05	0.66 ±0.05	0.67 ±0.05	0.66 ±0.05	0.66 ±0.04	0.65 ±0.05	0.66 ±0.06
	Phase III	0.52 ±0.05	0.54 ±0.05	0.53 ±0.05	0.50 ±0.05	0.52 ±0.04	0.53 ±0.06	0.54 ±0.05	0.53 0.06
reaction time – naming (s) {baseline}	Phase II	0.67 ±0.07	0.68 ±0.08	0.67 ±0.06	0.67 ±0.07	0.66 ±0.08	0.67 ±0.07	0.67 ±0.05	0.66 ±0.05
	Phase III	0.56 ±0.08	0.57 ±0.08	0.58 ±0.10	0.55 ±0.09	0.56 ±0.07	0.55 ±0.07	0.57 ±0.08	0.56 0.09
reaction time – reading (s) {interference}	Phase II	0.78 ±0.10	0.79 ±0.10	0.79 ±0.09	0.78 ±0.10	0.80 ±0.08	0.79 ±0.09	0.79 ±0.11	0.79 ±0.10
	Phase III	0.62 ±0.10	0.64 ±0.10	0.64 ±0.10	0.60 ±0.09	0.62 ±0.07	0.64 ±0.09	0.65 ±0.10	0.63 ±0.10
reaction time – naming (s) {interference}	Phase II	2.76 ±3.15	2.78 ±3.10	2.76 ±2.78	2.74 ±3.13	2.75 ±3.01	2.74 ±3.15	2.73 ±2.70	2.72 ±2.01
	Phase III	2.25 ±2.70	2.26 ±2.70	2.25 ±2.72	2.22 ±2.71	2.25 ±2.70	2.02 ±2.46	2.26 ±2.71	1.67 ±1.68

Variables are shown as mean values ± standard deviation (SD).

Phase: II – immediately after the exercise, III – immediately after the vibration treatment, IV - 24 h after the vibration treatment; Frequency of vibrations: I: 2–52 Hz, II: 82–100 Hz; vibrotherapy duration: T1: 10 min, T2: 45 min; body position: A: Lying down with lower limbs raised by 20°, B: lying down.

The type of frequency used, position, and duration of the treatment did not play a statistically significant role in changing STROOP test results (*p* > 0.05).

## 4 Discussion

Prolonged physical exertion is a powerful stressor that alters a functional and emotional state of the human body (Tyka et al., 2018; Jahangiri et al., 2019; Armstrong et al., 2022). There has been currently great interest in the problem of how quickly the body is able to return to its optimal psychophysical state after exercise, which is particularly important in the restrictive professional training of athletes. This is important not only to restore athlete’s ability to perform effectively in a short period of time, but also to prevent possible burnout (Silva, 1990; Henschen, 2001; Cresswell and Eklund, 2005; Silva et al., 2022). Prioritizing sleep, rest, nutrition, hydration, and joint range of motion during the recovery phase is fundamental, and then consideration should be given to restorative interventions that alleviate the specific physiological stress arising at any point in the recovery continuum (Jäger et al., 2008; Kellmann et al., 2018). To support mental regeneration, athletes introduce psychological techniques into their training routine, such as: progressive muscle relaxation (Battaglini et al., 2022), mindfulness (Coimbra et al., 2021) imagery (Di Corrado et al., 2020), awareness, positive self-talk and acceptance of pain as part of the sport (Kress and Statler, 2007), but they also use other methods, such as massage, which is considered the most effective alternative to psychological methods (Dupuy et al., 2018). However, the athlete does not always have the time or specialist help to undergo all the suggested psychological and rehabilitation procedures. Therefore, alternative holistic methods are being sought that would achieve satisfactory results in terms of both mental and physical regeneration. Vibration treatments appear to be one such method. It is worth highlighting that such treatments should be conducted under the supervision of specialists who choose the appropriate frequency, position, and duration (Jordan et al., 2005; Musumeci, 2017; Freitas et al., 2022).

The findings of this study are consistent with the ones of other researchers who reported positive effects of vibration treatments on emotional states, subjective pain sensations (Boucher et al., 2015; Maddalozzo et al., 2016; Dong et al., 2019), mental discomfort, and concentration (Regterschot et al., 2014).

In response to our question as to which frequency is more conducive to improvements, it was found that low-frequency treatments (2–52 Hz) promote greater improvements in mood, particularly in the vigor dimension. Although some other authors consider vibration frequencies between 20 and 70 Hz to be safe [e.g., Plentz and Sisto, 2018 as cited in Freitas et al. (2022)], we advise a caution to be exercised regarding far-reaching conclusions and further observations are suggested.

Regarding the question of whether the duration of treatment causes any changes to perceived pain, mental discomfort, or emotional states, it was only confirmed in the pain sensations dimension. The treatments of shorter duration (10 min) were more conducive to pain reduction than 45-min treatments. Previous studies on vibrotherapy showed that such treatments affect hormone levels and lymphatic drainage, leading to a reduction in pain and an improvement in mood state (Uher, 2018). Thus, based on the results from this study, it can be concluded that a 10-min treatment is completely sufficient to reduce pain sensations – this is probably due to the phenomenon of “gating” (Melzack and Wall, 1965; Moayedi and Davis, 2013), where under the influence of vibration, neurotransmitters are depleted in a relatively short period of time (Uher, 2018). The differences found in this study can be explained by the participants’ subjective perception of pain. It can be speculated that in both the 10-min and 45-min treatments, the perception of pain is most dynamic in the first minutes of the treatment (when gating occurs), which means that when the measurement was conducted at a shorter interval (10 min compared to 45 min), the subjects perceived the change to a greater extent, and as a result indicated lower post-treatment scores. However, in the 45-min measurement, it is possible that the subjects became accustomed to a normal state (normal pain level).

The last question concerned whether the position during the treatment causes any changes to perceived pain, mental discomfort, or emotional states. In this case no statistically significant differences were found.

The findings show that the best results related to mental recovery after intensive physical activity are obtained from vibrotherapy treatments of frequency (2–52 Hz), conducted in any position, and lasting 10 min. It should be noted here that such a treatment affects both the psyche and the somatics (Uher, 2018). This study has confirmed that vibrotherapy affects both the somatics, as indicated by numerous studies (Otsuki et al., 2008; Morel et al., 2017; Musumeci, 2017; Tyka et al., 2018; Piotrowska et al., 2021; Pałka et al., 2023) as well as the psyche. Thus, vibrotherapy can be considered as having a comprehensive effect.

This study focused only on selecting the optimal frequency, body position and duration of treatments aimed at accelerating post-exercise mental regeneration. Therefore, we did not include control measurements in our research, which is a certain limitation of our research. We would suggest that future research should compare the effectiveness of vibration treatments to control group and other methods that accelerate regeneration after intense physical exercise, such as: massage, intensive cooling, progressive muscle relaxation, or mindfulness. This would allow us to assess the usefulness of using vibration treatments as a holistic alternative to help return to homeostasis after intense exercise.

## 5 Conclusion

Findings from vibrotherapy research might have implication for competitive sports, as the new, effective ways of recovery after trainings and competition are being sought (e.g., intensive cooling, pneumatic sleeves for improving venous and lymphatic circulation). Vibrotherapy offers a chance to positively affect athletes’ physiological and psychological states such as their well-being and cognitive processes. This research enabled us to increase expand our knowledge of on this rarely discussed subject. Based on the results, it can be concluded that from psychological point of view, it is more effective to use 10-minute treatments at lower frequencies (in the range of 2–52 Hz).

## Data availability statement

The datasets presented in this article are not readily available because the data are not publicly available due to the reason: in accordance with the Personal Data Protection Act applicable in Poland, not all research participants have provided consent for the dissemination of their psychological research results. Requests to access the datasets should be directed to krzysztof.wrzesniewski@awf.krakow.pl.

## Ethics statement

The studies involving humans were approved by the Bioethics Committee of the PMWSZ in Opole No. KB/56/N02/2019. The studies were conducted in accordance with the local legislation and institutional requirements. The participants provided their written informed consent to participate in this study. Written informed consent was obtained from the individual (s) for the publication of any potentially identifiable images or data included in this article.

## Author contributions

KW: Conceptualization, Data curation, Formal analysis, Investigation, Methodology, Validation, Visualization, Writing – original draft, Writing – review & editing. TP: Resources, Writing – review & editing. JB: Conceptualization, Funding acquisition, Methodology, Project administration, Resources, Supervision, Validation, Writing – review & editing.
